# Assessing the Feasibility of Neutralizing Osteopontin with Various Therapeutic Antibody Modalities

**DOI:** 10.1038/s41598-018-26187-w

**Published:** 2018-05-17

**Authors:** Vahid Farrokhi, Jeffrey R. Chabot, Hendrik Neubert, Zhiyong Yang

**Affiliations:** 1 Biomedicine Design, Worldwide Research and Development, Pfizer Inc., Andover, Massachusetts, 01810 USA; 2 Biomedicine Design, Worldwide Research and Development, Pfizer Inc., Cambridge, Massachusetts, 02139 USA; 3 Inflammation and Immunology Research Unit, Worldwide Research and Development, Pfizer Inc., Cambridge, Massachusetts, 02139 USA

## Abstract

Osteopontin is a secreted glycophosphoprotein that is highly implicated in many physiological and pathological processes such as biomineralization, cell-mediated immunity, inflammation, fibrosis, cell survival, tumorigenesis and metastasis. Antibodies against osteopontin have been actively pursued as potential therapeutics for various diseases by pharmaceutical companies and academic laboratories. Many studies have demonstrated the efficacy of osteopontin inhibition in a variety of preclinical models of diseases such as rheumatoid arthritis, cancer, nonalcoholic steatohepatitis, but clinical utility has not yet been demonstrated. To evaluate the feasibility of osteopontin neutralization with antibodies in a clinical setting, we measured its physiological turnover rate in humans, a sensitive parameter required for mechanistic pharmacokinetic and pharmacodynamic (PK/PD) modeling of biotherapeutics. Results from a stable isotope-labelled amino acid pulse-chase study in healthy human subjects followed by mass spectrometry showed that osteopontin undergoes very rapid turnover. PK/PD modeling and simulation of different theoretical scenarios reveal that achieving sufficient target coverage using antibodies can be very challenging mostly due to osteopontin’s fast turnover, as well as its relatively high plasma concentrations in human. Therapeutic antibodies against osteopontin would need to be engineered to have much extended PK than conventional antibodies, and be administered at high doses and with short dosing intervals.

## Introduction

Osteopontin is a secreted glycophosphoprotein that has been shown to play important roles in a wide range of biological and pathological processes, such as biomineralization, wound repair/fibrosis, tumorigenesis, and cancer metastasis. Osteopontin is also known to be an important pro-inflammatory cytokine with pleiotropic functions^1–6^. Secreted osteopontin signals through two different sets of integrins via its RGD domain and a cryptic ^162^SVVYGLR^168^ sequence adjacent to the RGD domain. It also signals through CD44 variants via its C-terminal fragment^2^. Cumulative evidence suggests an important role for osteopontin in the pathogenesis of several immune-related diseases, such as rheumatoid arthritis (RA), multiple sclerosis, systemic lupus erythematosus, Sjögren’s disease, and colitis^2^. More recently osteopontin has been implicated as a key player in the pathogenesis of NASH, a disease characterized by an accumulation of fat in the liver, along with inflammation, hepatocyte ballooning and hepatic fibrosis. It has been shown to directly promote liver fibrosis by acting on cells such as hepatic stellate cells and hepatic progenitor cells^7,8^. Osteopontin neutralization using either an aptamer or a polyclonal antibody abrogated the liver progenitor cell response and fibrosis in three different mouse models of liver injury^9^. Because of its critical role multiple diseases, osteopontin has been widely explored as a therapeutic target in many preclinical studies^9–20^, as well as in a clinical trial^21^.

Several monoclonal antibodies against osteopontin have been reported, demonstrating protective efficacy in animal models of various diseases^11,12,15–17,19,22,23^. One such example is C2K1, a chimeric antibody which specifically recognizes the human osteopontin epitope, SVVYGLR. This antibody has been shown to ameliorate the established collagen-induced arthritis in cynomolgus monkey^15^. A similar antibody ASK8007 (Astellas Pharma Inc.) recognizes the same epitope and inhibits RGD as well as α_9_β_1_ integrin-dependent cell binding to human osteopontin. ASK8007 was evaluated in a double-blind, multi-center, combined first-in-man, single-dose escalation (phase I, part A) and proof-of-concept, multiple-dose (phase IIA, part B) study, in RA patients with active disease^21^. Results from this trial show that ASK8007 is overall safe and well-tolerated up to the highest studied dose (20 mg/kg). However, no clinical improvement was observed in the ASK8007-treated group in RA patients. As expected, administration of ASK8007 led to an accumulation of full length osteopontin levels in plasma, caused by increased stability of antibody-antigen complex. Because the study did not measure the free concentrations of osteopontin in the plasma, it is possible that the lack of efficacy in this study was due to insufficient target coverage, although other reasons cannot be excluded. For example, thrombin-cleaved osteopontin fragment, OPN-R, is believed to play more important roles in RA pathogenesis than the full-length osteopontin^24^. The effects of ASK8007 treatment on OPN-R were not measured.

Another monoclonal antibody developed against osteopontin is AOM1 (Pfizer Inc.). AOM1 is a fully human IgG2 which was identified using phage display technology. It recognizes the SVVYGLRSKS sequence which spans the thrombin cleavage site of the human osteopontin. AOM1 efficiently inhibited osteopontin binding to recombinant integrin α_v_β_3_ with an IC_50_ of 65 nM. This antibody was evaluated in a metastatic model of non-small cell lung adenocarcinoma (NSCLC), the Kras^G12D-LSL^p53^fl/fl^ GEMM (genetically engineered mouse model). Treatment of tumor bearing mice with AOM1 as a single agent or in combination with carboplatin significantly inhibited growth of large metastatic tumors in the lung, supporting a role for osteopontin in tumor metastasis and progression^19^.

We sought to evaluate the feasibility of developing a therapeutic antibody that can inhibit osteopontin-mediated events and related disease pathology in patients. To better understand osteopontin as a therapeutic target and the likely outcome after antibody treatment in human, we performed preliminary pharmacokinetic (PK)/pharmacodynamic (PD) modeling and simulation. Even heavily simplified models can be useful for prioritizing targets and experiments in an early stage portfolio, before investing effort and expense in more in-depth explorations to uncover further biology and to parameterize a more detailed model. One critical parameter for these preliminary models is the turnover of the target, as the quantity of drug dosed must be adequate to “keep up” with the production of target (its abundance multiplied by its turnover rate) over a dosing interval. While the circulating concentrations of potential targets such as osteopontin can frequently be found in the literature, estimates of turnover rates, especially in human subjects, are much scarcer. We therefore measured the physiological fractional synthesis and clearance rates of osteopontin in serum of human healthy subjects by using a stable isotope labeled *[5*,*5*,*5-*^2^*H*_3_*]-leucine* (D_3_-leucine) pulse-chase method, combined with immunoaffinity enrichment (IA) and liquid chromatography-tandem mass spectrometry (IA-LC-MS/MS) analysis^25,26^. Modeling and simulation was performed to compare different theoretical scenarios using the measured physiological turnover rate, to estimate the reduction in plasma levels of free osteopontin in human treated with various doses of antibodies. To expand the set of possible therapeutic solutions, antibodies with different properties and much improved PK (pH dependent antigen binding; active uptake by FcRn-expressing cells) were explored using the model.

## Materials and Methods

### Materials

Labeled microbiological and pyrogen tested L-leucine (5,5,5-^2^H_3_, 99%) for human IV injection was purchased from Cambridge Isotope Laboratories (Andover, MA). Antigen affinity-purified polyclonal human osteopontin antibody (goat IgG, AF1433) was purchased from R&D Systems (Minneapolis, MN). Dithiothreitol (DTT), iodoacetamide (IAA) and EZ-Link Sulfo-NHS-LC-Biotin and Zeba size exclusion 7 K MWCO 0.5 mL spin desalting columns were purchased from Thermo (Rockford, IL). Mass spectrometry grade trypsin/Lys-C mix was purchased from Promega (Madison, WI).

### Human D_3_-leucine pulse-chase study

Healthy human volunteers were recruited for the study at Clinical Pharmacology of Miami (CPMI). The study was with the informed written consent from the volunteers and approved by Western Institutional Review Board (Olympia, Washington, USA). All experiments and analysis in this study were performed in accordance with relevant guidelines and regulations. As described previously^26^, three healthy human volunteers were administered by intravenous infusion with a prime dose of 1.3 mg/kg body weight of a D_3_-leucine followed by 18-hour infusion at a constant rate of 0.022 mg/kg body weight per minute. In the span of 36 hours of study, 24 blood samples were collected longitudinally. Half of the samples were collected after termination of infusion. Blood samples were processed to generate serum and stored at −80 °C.

### Anti-osteopontin antibody biotinylation

As described in detail^26^, 2 µL of a 10 mM EZ-Link Sulfo-NHS-LC-Biotin solution in phosphate buffered saline (PBS) pH 7.2 was added to 100 µL of a solution containing 1 µg/µL anti-osteopontin antibody (see Materials). The mixture was incubated at room temperature for 2 hours. 2 µL of 1 M Tris HCl was added to scavenge the free biotin reagents. Size exclusion spin columns were used to separate biotinylated antibody.

### Immunoaffinity enrichment of osteopontin and protein digestion

**Figure 1:**
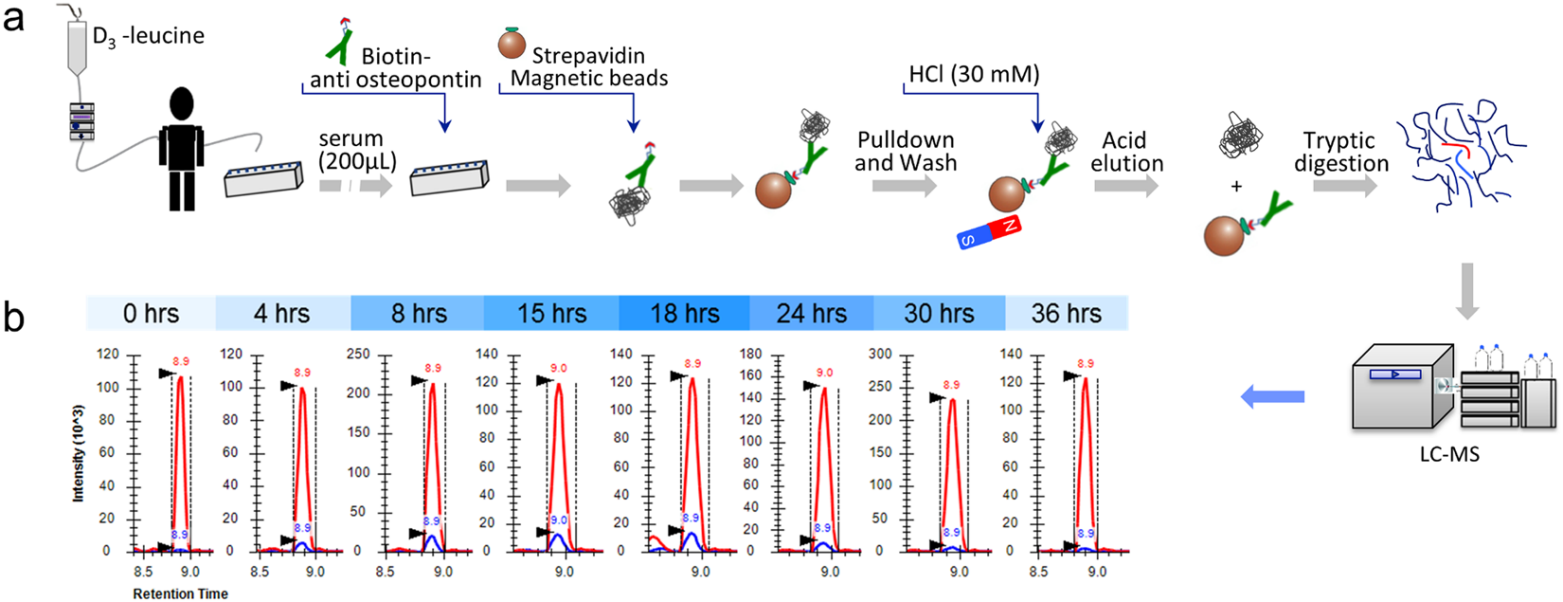
(**a**) Workflow for measurement of physiological protein turnover rate in human. A stable isotope labeled (D_3_-leucine) pulse-chase was combined with immunoaffinity enrichment and liquid chromatography-tandem mass spectrometry (IA-LC-MS/MS) analysis. (**b**) Chromatograms show the MRM signal of heavy (blue) and light (red) peptide (GDSVVYGLR) of osteopontin at selected time points.

Based on the previously developed serial immunoaffinity enrichment workflow^26^, supernatant of a serum sample set that was used for immunoaffinity enrichment of other unrelated proteins, was subsequently utilized for enrichment of osteopontin. As shown in Fig. 1a, an aliquot of 200 µL of each serum sample was diluted with addition of 400 µL of phosphate-buffered saline with 0.05% tween 20 (CMF-PBST), pH 7.2. Total amount of 1 µg (10 µL of 0.1 µg/µL) biotinylated anti-osteopontin antibody was added to each sample in a 96 deep well plate and incubated overnight at 4 °C. 20 µL of MyOne Streptavidin T1 Dynabeads, previously washed with PBST, were added to each sample and the sample plate was incubated on a mixer (800 rpm) at ambient temperature for 45 minutes. Automated pulldown and elution steps were performed on a Hamilton liquid handler (Microlab Star, Bonaduz, Switzerland) as illustrated in Fig. 1a^27–29^. Beads were washed twice with PBST and once with PBS to remove residual detergent. Bound proteins were eluted from beads with addition of 140 µL of 30 mM HCl and 30 µL of 1 M Tris HCl was added to each sample for pH neutralization. Eluted protein content was reduced with addition of DTT at a final concentration of 2 mM and incubated at 60 °C for 45 minutes. IAA at a final concentration of 4 mM was added and incubated for 45 minutes at dark for carbamidomethylation of the free cysteine residues. 10 µL of a 0.1 µg/µL trypsin/LysC mixture was added to each sample and incubated overnight at 37 °C for protein digestion.

### GC-MS measurement of D_3_-leucine in serum

Serum D_3_-leucine enrichment was measured by GC-MS as described in detail by Price *et al*.^30^. Briefly, after precipitation and removal of the protein content serum amino acids were derivatized with pentafluorobenzyl bromide and the leucine enrichment ratios were measured in each sample by gas chromatography-mass spectrometry (GC Agilent 6890 N with MS Agilent 5973) operated in NCI mode with a RTx-50 column (Restek, Bellefonte, PA).

### Nano flow liquid chromatography-tandem mass spectrometry

Protein digests were analyzed on a nano flow LC-MS/MS system. As previously described^26^, 50 µL of each sample was injected using an Ultimate 3000 (Dionex, Thermo) nano LC system linked to a TSQ-Quantiva™ triple quadrupole mass spectrometer (Thermo Scientific, USA) equipped with EASY-Spray™ Source for targeted analysis of peptides in multiple reaction monitoring (MRM) mode. A proteotypic and leucine-containing peptide from osteopontin (GDSVVYGLR) was selected for tracer enrichment analysis. Three transitions of y6, y5 and y4 from the doubly charged peptide were monitored from both peptide isotopologues (light and heavy). Acquired data were transferred to Skyline 3.5 and after peak integration, the heavy/light peptides peak area ratios were converted to heavy/total (light plus heavy) ratios. At time zero, interference from [M + 3] natural isotopic peak of the light peptide was expected and observed in the heavy channel. This interference is due to limited 3 Da mass shift that D_3_-leucine provides. A previously presented mathematical approach^26^ was taken to calculate and confirm the subtraction of this interference. The calculated interference (0.98%) was in agreement with average (1.05%) experimentally observed interferences at time 0 and was subtracted from all time points.

### PK/PD modeling and simulation of antibody ligand interactions in human

Quantitative dose-response pharmacokinetic/pharmacodynamics (PK/PD) models for potential anti-osteopontin antibody therapeutics were constructed as follows. For simplicity, a single compartment model with 5 L total volume (typical for large molecules in humans) was used. Osteopontin was initialized to a given steady state level (minimum 112 ng/mL, maximum 1740 ng/mL, and geometric mean 444 ng/mL)^31^, with first order clearance rate as measured in this work and constitutive production to maintain this level in the presence of this clearance. With a one-week dosing interval, a quantity of antibody is introduced into this compartment, which can reversibly bind to free osteopontin with k_on_ = 10^5^ M^−1^ s^−1^ (typical) and k_off_ = k_on_*K_d_, with K_d_ a proposed equilibrium dissociation constant (i.e., affinity).

The four potential therapeutic strategies are implemented as follows:

- Standard antibody: 17 day half-life for both antibody and antibody-OPN complex.
- Perfect pH switch: 17 day half-life for free antibody; 1 day half-life for complex (returning free antibody)^32^.
- Perfect “sweeper”: 17 day half-life for free antibody; 2 hour half-life for complex (returning free antibody) (estimated from the maximum antibody-driven turnover of target in for the most potent “v6-type” sweeper)^33^.
- Realistic “sweeper”: 2.25 day half-life for free antibody (estimated antibody half-life for v6-type sweeper); 2 hour half-life for complex (returning free antibody)^33^.

A value of 1 nM was used as the K_d_ for simulation as tighter binding would not lead to endosomal release with a reasonable change in affinity for the acid switch (~tenfold)^32^ and looser binding would not sufficiently engage osteopontin at neutral pH. In any case, the model predictions were only weakly sensitive to increasing affinity beyond this level (results not shown). The model output was the average free level of osteopontin over the one week dosing interval at steady state, expressed as a fraction of the initial osteopontin level. The equations and parameters of the various models are supplied in Supplementary Information.

## Results

### Mass spectrometry measurement of newly synthesized osteopontin

Immunoaffinity enrichment combined with LC-MS/MS detection of D_3_-leucine tracer incorporation into osteopontin derived tryptic peptides enabled the turnover measurement (Fig. 1a). The signal from newly synthesized peptide is immediately observable in the first time point sample after infusion start (2 hours) with high signal/noise ratio (Fig. 1b). The tracer incorporation profile followed closely the serum D_3_-leucine enrichment profile and were found to be between 1% and 13%. High analytical sensitivity enabled by targeted immunoaffinity enrichment of osteopontin and multiple reaction monitoring (MRM) MS analysis was critical for high confidence turnover measurement.

### Calculation of osteopontin physiological turnover rate in human

**Figure 2:**
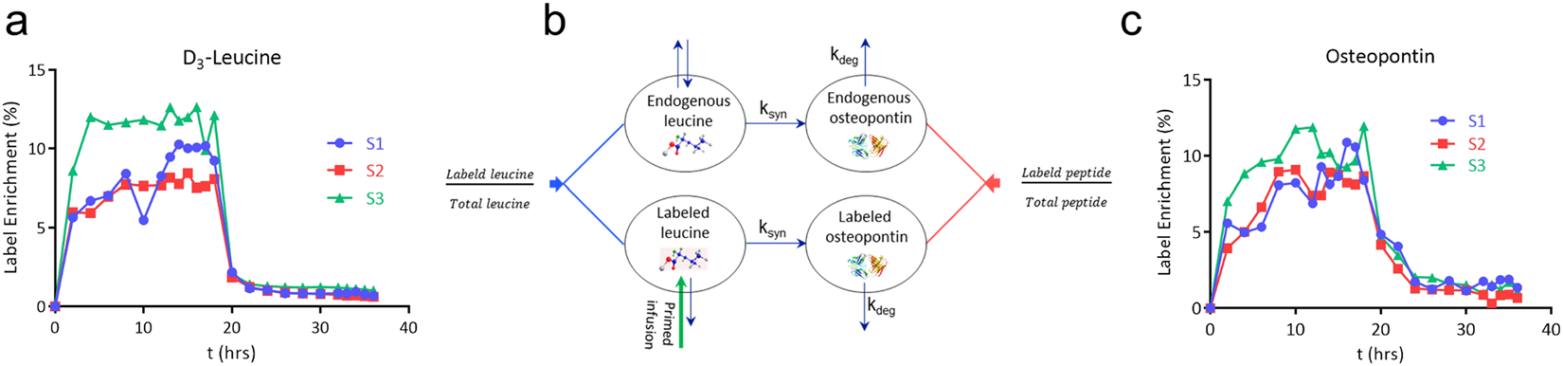
(**a**) D_3_-leucine enrichment profiles in serum of three human subjects. (**b**) Dynamic four compartmental PK model for osteopontin half-life estimation. (**c**) Time course of labeled leucine enrichment in osteopontin peptide (GDSVVYGLR) during the study.

MS peak area ratios were exported into the SAAM II software (The Epsilon Group, University of Washington) where a four species PK model (Fig. 2b) was utilized to fit both leucine precursor (Fig. 2a) and osteopontin product (Fig. 2c) label enrichment data^34^. Osteopontin half-life was calculated from peptide GDSVVYGLR label enrichment results on the basis of first order kinetics (t_1/2_ = ln(2)/k_deg_). As can be seen in Figs 1b and 2c, the newly synthesized peptide that carries the heavy leucine tracer, immediately appears in the first time point after start of the infusion and quickly reaches a plateau in all three subjects. When compared to the leucine tracer enrichment profile in serum, it is clear that the label enrichment in osteopontin almost immediately reaches levels close to the precursor. This clearly implies a very fast fractional synthesis rate of osteopontin and its appearance in the circulation. With steady state assumption in which synthesis (k_syn_ × Pre_0_, synthesis constant times precursor concentration) is equal to the degradation of the target (k_deg_ × Pro_0_ degradation constant times target concentration), a fast clearance of osteopontin also is expected. Indeed, after 18 hours, when the infusion of the tracer is terminated and levels of labeled leucine decrease close to baseline (Fig. 2a), labeled osteopontin also decreases quickly following a trend similar to serum leucine profile, implying a very fast fractional clearance rate of osteopontin. Both pulse and chase phases of the study confirm a fast turnover rate for osteopontin in serum from healthy human volunteers. The calculated half-life of osteopontin from the fitted data was 11 minutes (an average of t_1/2_ values determined in 3 subjects: 15, 11, 7 minutes).

### PK/PD modeling and simulation of antibody ligand interactions in human with various scenarios

**Figure 3:**
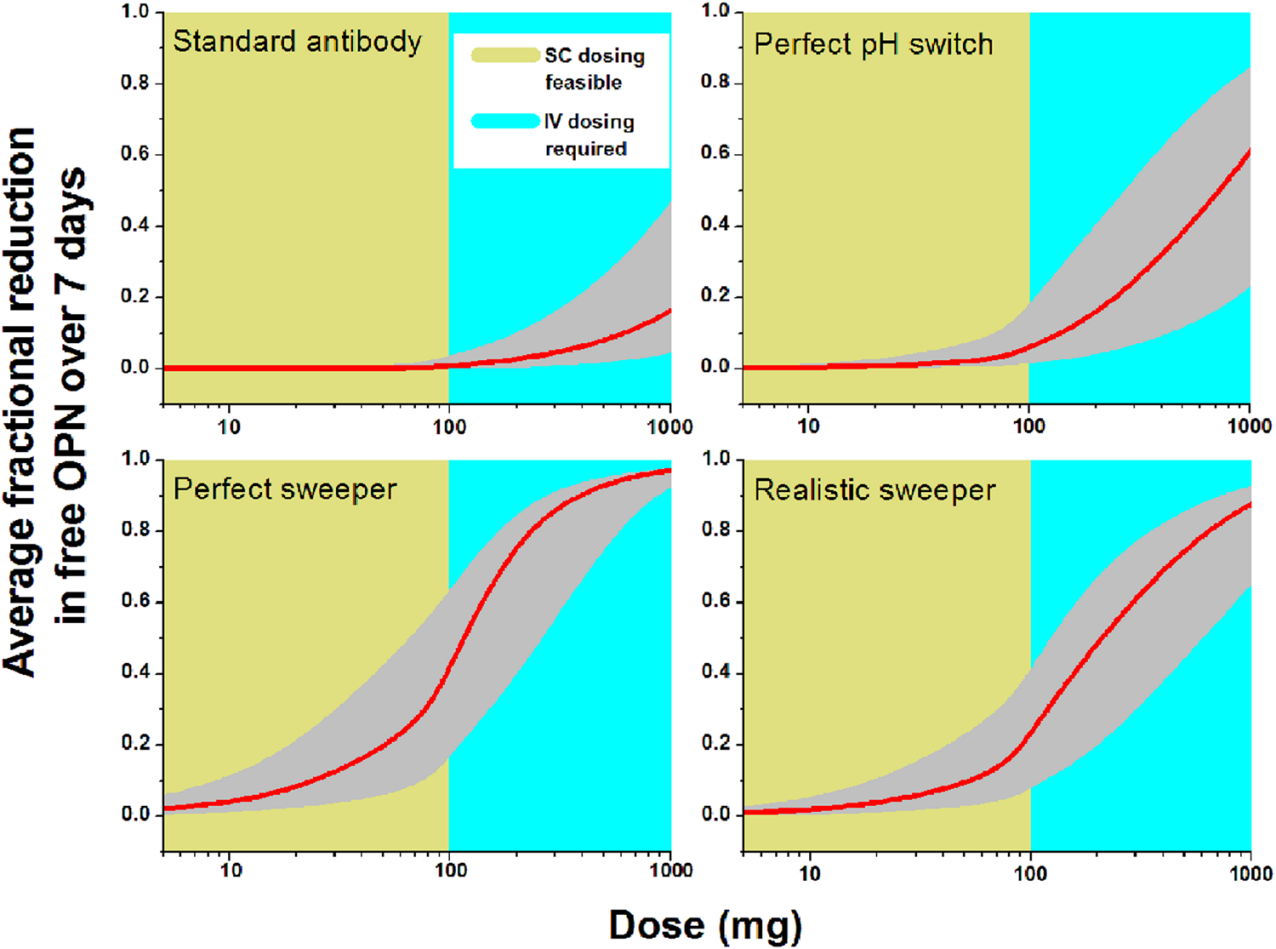
Simulations illustrating effects of varying antibody doses and dosing intervals in an antibody-ligand PK/PD model.

Based on the turnover data and other biomeasure information, we constructed quantitative dose-response pharmacokinetic/pharmacodynamics (PK/PD) models for potential anti-osteopontin antibody therapeutics as described in Methods. It is clear that achieving sufficient target coverage using standard antibodies would not be feasible in human, mostly due to the fast turnover of osteopontin, as well as its high plasma concentrations (Fig. 3). The degree of knockdown depends on the starting osteopontin level. The shaded region reflects the range of osteopontin levels in human, based on literature data (112–1740 ng/mL)^31^, while the solid line represents the geometric center of this range (444 ng/mL). The model predicts that weekly administration of a standard antibody at doses as high as 1000 mg can only cause ~20% reduction in free osteopontin concentration. Doses up to 100 mg are typically achievable using single injection subcutaneous (SC) delivery. A bioavailability of 50% was assumed for these doses. Doses greater than 100 mg generally require intravenous (IV) dosing.

Antibody engineering technologies exist, which can significantly extend antibody serum half-life. The variable region can be engineered such that an antibody can bind to an antigen in plasma at neutral pH, whereas the antibody-antigen complex dissociates at the endosomal pH of 5.5–6.0 to allow the antibody to escape from target-mediated degradation (pH switch). Further, the constant region can be engineered to increase the neonatal Fc receptor (FcRn)-mediated cellular uptake of the antibody-antigen complex into endosome^33^. This type of antibody can be called a “sweeper” since it increases the elimination of soluble antigens from circulation. In our simulation, four therapeutic scenarios were contemplated using parameters described in the Methods to compare the treatment effects on osteopontin using a standard antibody, a perfect pH switch, a perfect sweeper or a realistic sweeper. Figure 3 shows the predicted average free osteopontin reductions for each of the four scenarios for weekly (QW) dosing. The “perfect sweeper” was able to achieve greater than 40% average osteopontin knockdown over the 1 week dosing interval with subcutaneous dosing. However, this hypothetical construct is likely impractical to produce, as the sweeping mechanism by its nature causes the therapeutic to be taken up and processed with a much higher rate than a standard antibody. Therefore, a sweeper antibody is usually accompanied by a greater opportunity for clearance by failure to recycle. The more realistic sweeper with high doses (1000 mg), frequent (weekly) IV administration was able to produce an average of 90% knockdown in free osteopontin levels. However, while such dosing is possible, the high amount of drug substance and inconvenience of administration generally precludes such a regimen except in extreme cases such as aggressive cancer therapeutics (e.g. up to 500 mg/m^2^ for rituximab, up to 400 mg/m^2^ for cetuximab, up to 15 mg/kg for bevacizumab) or a few other select indications (e.g. up to 1200 mg for eculizumab for hemolytic uremic syndrome).

## Discussion

PK/PD modeling and simulation of antibody-ligand interactions *in vivo* have become increasingly useful in development of therapeutic antibodies. This exercise helps predict the required antibody properties including its binding affinity, PK, dosage and dosing intervals. To better understand antibody effects on an endogenous ligand *in vivo*, some key parameters, such as the physiological turnover rate and its concentration in plasma need to be determined. In this study we focused on the understanding of ligand modulation, by assuming that free osteopontin unbound to an antibody is what drives pharmacology. We used single compartment model for simplicity, assuming that plasma is the “site of action” for antibody (best-case scenario for effective osteopontin binding). We used a stable isotope (D_3_-leucine) pulse-chase tracing method, combined with immunoaffinity enrichment (IA) and liquid chromatography-tandem mass spectrometry (IA-LC-MS/MS) analysis, and determined that osteopontin undergoes rapid turnover in heathy human subjects (average t_1/2_ is ~11 minutes). Using the same set of longitudinal serum samples collected from 3 healthy human subjects, we recently performed turnover analysis of a few other proteins including soluble tumor necrosis factor receptor superfamily member 12 A (sFN14), tissue factor pathway inhibitor (TFPI), soluble interleukin 1 receptor like 1 (sST2), and muscle-specific creatine kinase (CKm). Calculated half-lives for these 4 proteins ranged from 5 to 15 hours^26^. By comparison, the turnover rate of osteopontin is unusually fast. PK/PD modeling and simulation suggest that a standard antibody with high-affinity administered at a reasonable dose and interval would not appreciably knock down plasma osteopontin levels in human. Antibodies can be engineered (pH-dependent recycling antibodies, or sweeping antibodies with additional Fc engineering) to dramatically extend PK and to eliminate the antigen from plasma, affording huge reduction of the antibody dosage and dosing frequency needed for efficacy^33^. Simulations show that, even for these types of antibodies, sufficient target coverage remains a substantial challenge, mostly due to its very rapid turnover rate. High-dose IV infusion of such antibodies with very short dosing intervals would likely be required in order to effectively inhibit osteopontin activity. Our results may help explain why anti-OPN mAb ASK8007 failed to show efficacy in RA patients in the clinical trials.

Generally, highly simplified models may be useful early in the drug discovery process for portfolio management, prioritizing potential targets and/or selecting modalities. For feasibility assessments, modeling “best-case” scenarios is often appropriate, as a projected failure to achieve the desired endpoints would be unlikely to be corrected with a more accurate and detailed model, and can save the time and expense associated with constructing and parameterizing such a model. Conversely, strategies predicted to be successful with these models may not fare well once more realistic conditions are introduced. It should be noted that many of the implicit assumptions in the antibody simulation scenarios represent the most optimistic cases for achieving high levels of osteopontin binding; for example, using circulating concentrations rather than higher local “site of action” concentrations, and assuming that unloading of pH switch/sweeper antibodies occurs with perfect efficiency. These assumptions should be challenged in a more detailed model (supported with preclinical validation) used to make efficacious dose projections for an actual clinical candidate molecule, but serve a useful role in early discovery programs to prioritize targets and experiments when a limited dataset is available for informing such a model and with minimal commitment of additional resources.

## Electronic supplementary material

Supplementary Information
