# Supplementary material for: Assessing the Feasibility of Neutralizing Osteopontin with Various Therapeutic Antibody Modalities
Source: Sci Rep. 2018 May 17;8:7781. doi: 10.1038/s41598-018-26187-w (PMC5958109; doi:10.1038/s41598-018-26187-w)
Supplement: Supplementary file 1 — Supplementary Information [file 41598_2018_26187_MOESM1_ESM.docx]

**Assessing the Feasibility of Neutralizing Osteopontin with Various Therapeutic Antibody Modalities**

**Vahid Farrokhi, Jeffrey R. Chabot, Hendrik Neubert, and Zhiyong Yang**

**Supplementary Information**

**Model equations and parameters**

State variables *[X]* are expressed as concentrations.

**Equations for “standard antibody” PK/PD model:**

$$\frac{d}{dt}\left[ OPN \right]=k_{synth,OPN}-k_{on}\cdot\left[ OPN \right]\cdot\left[ antibody \right]+k_{off}\cdot\left[ complex \right]-\frac{\ln\left( 2 \right)}{t_{\frac{1}{2},OPN}}\cdot[OPN]$$

$$\frac{d}{dt}\left[ complex \right]=k_{on}\cdot\left[ OPN \right]\cdot\left[ antibody \right]-k_{off}\cdot\left[ complex \right]-\frac{\ln\left( 2 \right)}{t_{\frac{1}{2},antibody}}\cdot[complex]$$

$$\frac{d}{dt}\left[ antibody \right]=dose\left( t \right)-k_{on}\cdot\left[ OPN \right]\cdot\left[ antibody \right]+k_{off}\cdot\left[ complex \right]-\frac{\ln\left( 2 \right)}{t_{\frac{1}{2},antibody}}\cdot[antibody]$$

**Equations for “perfect pH switch” and “sweeper” PK/PD models:**

$$\frac{d}{dt}\left[ OPN \right]=k_{synth}-k_{on}\cdot\left[ OPN \right]\cdot\left[ antibody \right]+k_{off}\cdot\left[ complex \right]-\frac{\ln\left( 2 \right)}{t_{\frac{1}{2},OPN}}\cdot[OPN]$$

$$\frac{d}{dt}\left[ complex \right]=k_{on}\cdot\left[ OPN \right]\cdot\left[ antibody \right]-k_{off}\cdot\left[ complex \right]-\frac{\ln\left( 2 \right)}{t_{\frac{1}{2},antibody}}\cdot\left[ complex \right]-\frac{\ln\left( 2 \right)}{t_{\frac{1}{2},pH switch}}\cdot[complex]$$

$$\frac{d}{dt}\left[ antibody \right]=dose\left( t \right)-k_{on}\cdot\left[ OPN \right]\cdot\left[ antibody \right]+k_{off}\cdot\left[ complex \right]-\frac{\ln\left( 2 \right)}{t_{\frac{1}{2},antibody}}\cdot\left[ antibody \right]+\frac{\ln\left( 2 \right)}{t_{\frac{1}{2},pH switch}}\cdot[complex]$$

**Parameters:**

| **Model specific parameters** | **Standard** | **pH switch** | **Perfect sweeper** | **Realistic sweeper** |
| --- | --- | --- | --- | --- |
| $t_{\frac{1}{2},antibody}$ | 17 days | 17 days | 17 days | 2.25 days |
| $t_{\frac{1}{2},pH switch}$ | N/A | 1 day | 2 hr | 2 hr |
|  | | | | |
| **Shared parameters** | | | | |
| $Dose(t)$ | $\frac{Antibody dose (mg)\cdot bioavailability}{{MW}_{antibody}\cdot Volume}\cdot{MW}_{antibody}$  (added as bolus at time of dosing; 0 otherwise) | | | |
| $Volume$ | 5 L | | | |
| $bioavailability$ | 0.5 (for subcutaneous dosing), 1 (for intravenous dosing) (dimensionless) | | | |
| ${MW}_{antibody}$ | 150 kDa | | | |
| $k_{synth,OPN}$ | ${[OPN]}_{baseline}\cdot\frac{ln(2)}{t_{1/2,OPN}}$ | | | |
| $t_{1/2,OPN}$ | 11 minutes | | | |
| ${[OPN]}_{baseline}$ | 112-1740 ng/mL (3.5-54.4 nM), geometric mean 444 ng/mL (13.9 nM)  (MW_OPN_ = 32 kDa) | | | |
| $k_{on}$ | 100000 M^-1^ s^-1^ | | | |
| $K_{d}$ | 1 nM | | | |
| $k_{off}$ | $k_{on}\cdot K_{d}$ = 10^-4^ s^-1^ | | | |
